# Effect of an Inert Gas Positive-Pressure Environment on Beryllium Melting under a Pulsed Laser

**DOI:** 10.3390/ma15144916

**Published:** 2022-07-14

**Authors:** Yuxin Sang, Muzheng Xiao, Zhijing Zhang, Jiangzhou Su

**Affiliations:** 1 School of Mechanical Engineering, Beijing Institute of Technology, Beijing 100081, China; sangyx0825@163.com; 2 Beijing Hangxing Machinery Manufacture Limited Corporation, Beijing 100013, China; 13342261232@163.com

**Keywords:** positive pressure, single-pulse laser processing, beryllium, surface appearance, microstructure, cracks

## Abstract

Beryllium is widely used in the manufacturing of precision instruments because of its high thermal and mechanical properties. However, because beryllium is expensive, and processing it generally uses subtractive manufacturing methods, the cost is high, the utilization rate of cutting the materials is low, and the processing is difficult. Additionally, it is extremely prone to cracking, brittle fracturing, and fracturing during the machining process. In this paper, a new method for manufacturing beryllium laser additives under a pressure atmosphere is proposed. Via the single-point and single-pass laser melting of beryllium materials in an inert gas (Ar) pressure atmosphere, the results of the experiments conducted in the pressure range of 1 to 30 bar indicated the following: (1) beryllium can absorb the laser and form a molten pool, and the contour area of the upper surface of the molten pool is approximately 80% of that of 304 stainless steel under the same energy input; (2) severe oxidation occurs on and near the molten pool surface under low pressure, and oxidation is eliminated when the pressure is increased; (3) as ambient pressure increases, the surface profile of the molten pool gradually exhibits an irregular shape, and the cracks on the surface of beryllium change from “divergent” to “shrinkage”, which can eliminate cracking. At higher pressures, the “small hole” phenomenon in the molten pool disappears, forming a wide and shallow molten pool shape that is more conducive to stable deposition. The experimental results indicate that the laser-additive manufacturing of beryllium in a pressure atmosphere is a meaningful developmental direction for beryllium processing in the future.

## 1. Introduction

Beryllium is a structural material with many advantages, such as being lightweight and having high specific strength, high specific rigidity, stable dimensional accuracy, a thermal expansion coefficient matching that of steel, and nonmagnetic properties. It is one of the best structural materials for advanced products such as aircraft, missiles, rockets, spaceships, and nuclear submarines [1,2]. At the same time, beryllium is used as a plasma-facing material in the application and future development of fusion devices, and its role is very important [3,4]. However, the traditional manufacturing method (cutting) of beryllium materials encounters a series of problems. First, beryllium is an expensive metal. Generally, the more economical method is to let raw material suppliers provide blanks as close as possible to the final size of the workpiece; thus, machining manufacturers can reduce the amount of cutting in the subsequent machining process. The beryllium material is prepared using the hot isostatic pressing (HIP) of beryllium powder, which has a high cost and low utilization rate for cutting materials. Additionally, cutting beryllium materials is difficult. Beryllium is very brittle. Before machining, the workpiece must be clamped rigidly; otherwise, it breaks or cracks at the cutting entry and exit points. In the process of machining, brittle fracturing, edge slag falling, fracturing, and other phenomena occur very easily, resulting in scrapped parts and rapid tool wear, making it difficult to ensure consistency in machining accuracy [5,6,7,8,9]. Therefore, a more efficient and low-cost manufacturing method for beryllium materials is required.

Metal additive manufacturing technology can rapidly form parts with complex shapes, shorten the development cycle, reduce the development cost of parts, and manufacture parts with superior microstructures and mechanical properties. It has wide application prospects in industries such as aerospace, weapon equipment, and automobile complex parts [10,11,12,13,14,15]. In 2000, titanium alloy parts manufactured by the American company Boeing using laser solid forming were used in F-22 aircraft [10]. In 2015, NASA successfully tested a rocket engine using multiple 3D printed complex parts. The turbo pump, injectors, and valves of the rocket engine were manufactured using selective laser melting [15]. In recent years, well-known automobile manufacturers, such as BMW and Mercedes-Benz, have manufactured important automobile parts using metal additive manufacturing and have successfully applied them to mass-produced models.

However, current problems are also apparent. In essence, the manufacturing process of metal additives is also a process of rapid metal melting, solidification, and forming; therefore, it is difficult to avoid the existence of a large gradient and many thermal cycles, which can easily produce cracks, internal stress accumulation, coarse grains, and uneven growth defects and deficiencies. In addition, because the material is deposited and solidified layer by layer, oxides are inevitably left on the surface of each layer in the process of metal melting. These oxides remain in the deposited metal with the deposition of the next layer, resulting in internal impurities, which further affect the performance of the metal additive components. Therefore, it is currently difficult for the structural parts directly processed by additive methods to become key structural parts for load-bearing.

Generally, post-treatment processes, such as aging treatment and HIP, are required for metal-additive-manufactured parts used in current engineering. The mechanical properties after forming are often slightly higher than those of castings, and the mechanical properties after post-treatment can be equivalent to those of forgings [16]. However, the stability of metal-additive-manufactured parts is limited because of some common problems such as internal cracks, pores, composition segregation, and insufficient accuracy; thus, the yield often cannot satisfy the requirements of mass production.

The method of using pressure to improve the performance of metal-additive-manufactured parts primarily involves the post-treatment of the parts, i.e., HIP. The operating principle of HIP is to apply high temperature and high pressure to the parts simultaneously and maintain them for a certain time (the maximum pressure is between 100–200 MPa, and the temperature can reach 2000 °C) to eliminate defects such as cracks and pores produced in the casting of parts and increase the density of parts. Research shows that after HIP treatment, the fatigue strength of the parts is even higher than that of forgings, and the impact resistance is also significantly improved [17]. However, the HIP method has apparent limitations, such as its ability to effectively eliminate the closed hole, and it has no significant effect on the “tunnel type” defects extending to the surface of the component [18].

It is generally accepted that a pressure environment can improve the performance of metal-forming parts. However, through only HIP, the degree of optimization of the performance of the parts is limited, and the manufacturing time of the parts is extended to a certain extent, which covers the characteristics of efficient machining in additive manufacturing. Therefore, researchers can choose to exert external pressure during processing.

Both the Cranfield Welding Research Institute in the UK and Zhang Haiou at the Huazhong University of Science and Technology have proved that real-time rolling can aid in eliminating the residual stress of a component and transform the columnar dendrites in the deposition layer into equiaxed dendrites [19,20,21]. However, using a high-pressure roller to perform high-pressure treatment on components in real time has certain limitations. First, because the high-pressure roller occupies a certain volume, it can only roll along the deposition track, which is limited to the formation of complex-shaped components and can only assist in the deposition of simple thin-walled components; in contrast, the high-pressure roller is used to roll the metal after deposition, which primarily acts on the solid or semi-solid metal; therefore, a large pressure (above 20 kN) is required to produce the effect, which also demands higher requirements for the rolling equipment.

In the field of casting, the solidification structure can be improved by controlling the pressure in the forming process, primarily through vacuum casting and pressure solidification. In addition, in the field of welding, many methods of applying pressure to the environment have been reported. On one hand, this part of the research focuses on the performance control of the welding structure; on the other hand, it aims at understanding the application requirements of welding under a pressure environment.

In summary, metal hot-forming in a non-atmospheric pressure environment, particularly a positive pressure one (HIP, high-pressure roller, gas positive pressure, etc.), has many potential beneficial effects, such as eliminating residual stress, refining grains, and reducing defects. In terms of post-treatment, HIP cannot easily eliminate the large defects accumulated in the manufacturing process; in terms of process treatment, the use of a high-pressure roller is limited by the forming shape. Therefore, using gas pressure to create a positive pressure environment is a more continuous and uniform positive pressure-assisted manufacturing method. However, because customized high-pressure equipment is required for a manufacturing positive-pressure environment, most of the research on processing in a positive-pressure environment is focused on high-pressure welding, and the relatively higher requirements for laser cladding, additives, and other equipment have not been combined with positive-pressure environments. Additionally, previous research on non-atmospheric pressure welding was primarily focused on deep penetration welding. The influence of environmental pressure on the processing effect has not been reported in detail for laser additive manufacturing, which uses low energy density of high-energy beam processing conditions.

To solve the problem of metal additive manufacturing, this study focused on the method of using pressure to improve the metal solidification effect in traditional casting and welding processes and proposed a method of metal additive manufacturing in an inert gas positive-pressure atmosphere. Meanwhile, E Thoren et al. have proved that the modelling of shallow metallic melt layers within shallow water approximation is computationally efficient and very reliable in other physical fields [22]. With reference to this method, numerical modelling of this experiment can also be carried out. The base metal was placed in a customized small pressure vessel, and laser processing was performed through a quartz lens on the top of the pressure vessel. With this method, the additive manufacturing process is applied to an inert gas positive-pressure environment, and the metal melting and solidification are simultaneously subjected to a uniform gas atmosphere pressure. Many experiments have been performed on the basic problems of metal melting and solidification under different gas pressures, and comprehensive experimental data have been obtained, and preliminary cause analysis has been performed. The research results verify the beneficial effects of a gas positive-pressure environment on metal rapid-melting and solidification, such as inhibiting oxidation, eliminating defects, and refining grains. This creates a foundation for realizing additive manufacturing in a gas positive-pressure environment [23].

Laser melting experiments of beryllium (pure beryllium plate) were performed under normal and positive pressures to explore the feasibility of the lasered direct deposition of beryllium and the effects of an inert gas pressure environment in eliminating beryllium laser melting defects.

## 2. Experimental Setup

### 2.1. Workstation

**Figure 1:**
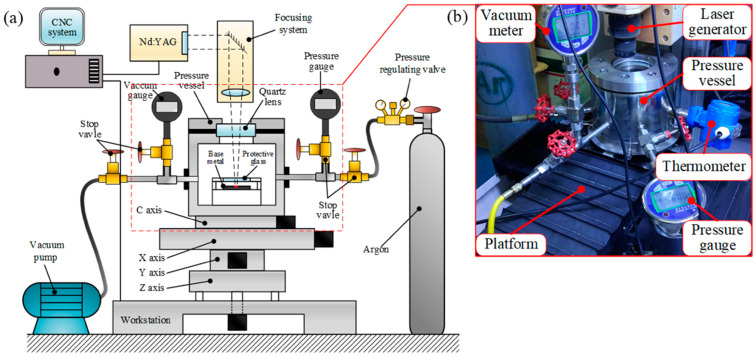
Inert gas positive-pressure vessel experimental device: (**a**) schematic diagram of the experimental setup, (**b**) physical diagram of pressure vessel.

The equipment used in this study was a closed high-pressure vessel device designed and built by the research team based on a self-developed composite processing machine (Figure 1). The main body of the tank was 200 mm in diameter and 210 mm in height. The tank was filled with argon to create an inert gas pressure environment. Before filling with argon, a vacuum pump with a limit vacuum degree of 2 Pa was used to vacuum the pressure tank. The wavelength of the laser beam was 1.06 μ. An Nd:YAG laser was generated in pulse mode and irradiated onto the base metal through the quartz lens on the pressure vessel. To collect the splashing metal particles produced by laser processing, the device set a collecting glass on the base metal to protect the quartz lens above.

In addition, the pressure vessel was connected to a vacuum gauge, pressure gauge, and four stop valves to monitor and regulate the pressure in the vessel. The entire pressure vessel was fixed on a four-axis worktable, and the worktable and laser were adjusted using a numerical control system.

### 2.2. Experimental Materials and Experimental Parameters

The material used in this experiment was a pure beryllium plate.

The relevant parameters used in this experiment are listed in Table 1 according to the summary of the previous research group’s experiment.

## 3. Experimental Program

### 3.1. Experimental Procedure

**Figure 2:**
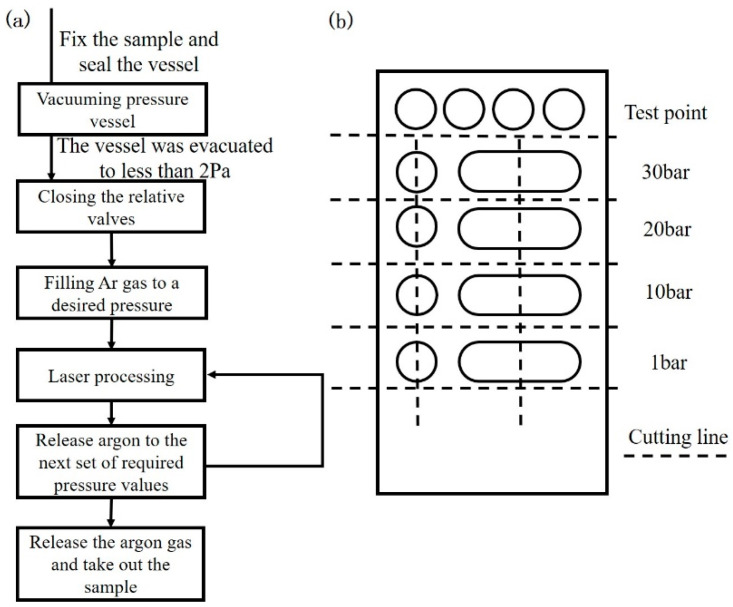
(**a**) Experimental procedure and (**b**) schematic of the laser processing scheme and parameters.

Figure 2 depicts the experimental process. As shown in Figure 2a, the beryllium plate was first fixed on the platform in the pressure vessel, and the pressure vessel was closed. The stop valve was closed on the side of the argon cylinder, and a mechanical vacuum pump was used to vacuum the pressure in the tank to less than 2 Pa. The stop valve was then closed at the vacuum pump, the vacuum pump was closed, the stop valve was opened at the argon cylinder, and argon was injected until the pressure reached 30 bar. The shut-off valve at the argon cylinder was closed, the gas pipe connecting the pressure vessel and argon cylinder was disconnected, and the opening of the gas pipe to the outside was connected (beryllium-containing steam was produced in the experiment, which required to be discharged to protect the safety of the test personnel). The laser was turned on, and laser processing was performed on the beryllium plate in the pressure vessel according to the laser parameters and motion table parameters listed in Table 1 under a 30 bar inert gas pressure atmosphere. The shut-off valve leaving the pressure vessel to the outside was opened, the gas in the vessel was gradually released to 20 bar, and the shut-off valve was closed. The laser processing experiment was repeated according to the set laser and motion table parameters. Similarly, laser processing experiments were performed under 10 and 1 bar environments. Finally, the samples were removed for subsequent testing.

Laser melting in laser processing is divided into single-point melting and single-pass melting (Figure 2b). Single-point melting is self-melting using a single pulse laser at each pressure; single-pass melting uses a 30 mm long-pulse laser self-melting under various pressures.

### 3.2. Observation and Test

To explore the influence of inert gas pressure on the surface morphology and microstructure of the laser processing melting pool, a series of material tests were performed in this study.

As shown in Figure 2b, metallographic sampling was performed with a wire cutting machine along a cross-section perpendicular to the scanning direction. In this study, the cold inlay method was used, and acrylic powder was used as the cold inlay powder, mixed with a special coagulant in a ratio of 1:1, poured into the mold where the sample was placed and cured for 25 to 30 min. The sample is ground and polished with a grinding and polishing machine. During the grinding and polishing process of the sample, the MTM-II microscopic visual inspection instrument is used for macroscopic observation on the millimeter scale, and the metallographic corrosion is carried out after confirming that the surface scratches are basically eliminated. The sample was electrolytically corroded with 10% oxalic acid solution, the voltage was 3 V, the current was 25 mA, and the corrosion time was about 30 s. After the corrosion was completed, the surface and cross-section of the sample were observed using a laser scanning confocal microscope (LSCM OLYMPUS-OLS4000). The surface morphology of the molten pool was determined using scanning electron microscopy (SEM). Additionally, the surface was scanned using energy dispersive spectroscopy (EDS) to obtain the qualitative composition ratio. Table 2 illustrates the above observation method, the corresponding observation position, and a schematic diagram.

## 4. Results

### 4.1. Surface Appearance of Molten Pools

**Figure 3:**
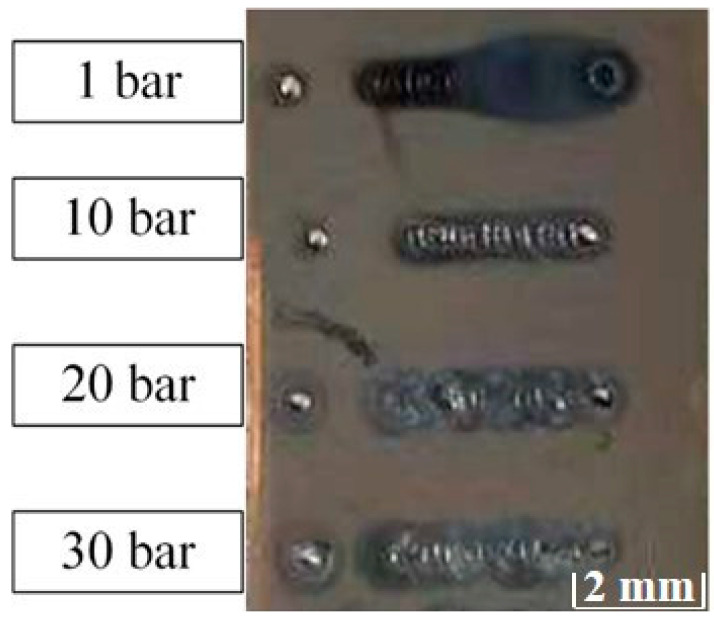
Macroscopic effect diagram of beryllium laser melting under different pressures.

**Figure 4:**
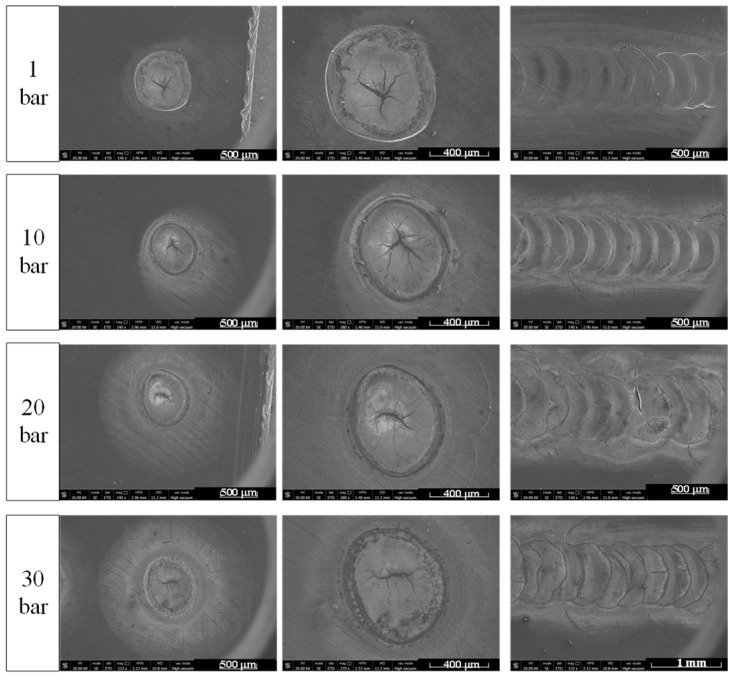
Surface effect diagram of beryllium laser melting under different pressures.

Figure 3 shows the macro effect images of laser melting, and Figure 4 shows the surface effect picture of laser melting. In the laser melting experiment of beryllium, the feasibility of the laser processing of beryllium was verified. Compared with stainless steel, which has the same energy density, beryllium can generally undergo laser melting and solidification, but because beryllium has a lower laser absorption [24], the overall surface profile area of the molten pool is reduced by approximately 20%.

Figure 3 shows that when the ambient pressure reached 10 bar or above, surface oxidation was eliminated.

As shown in Figure 4, when the pressure of the inert gas environment was greater than 10 bar, the surface began to appear irregular, meaning the point under each pulse melting was irregularly circular.

Cracks were also observed on the surface of the beryllium under the experimental energy density. With an increase in pressure, the surface cracks of beryllium change from “divergent” to “shrinkage”, which indicates that the high-pressure environment has the tendency to eliminate the cracks in the laser processing of beryllium.

### 4.2. Surface SEM/EDS Test

**Figure 5:**
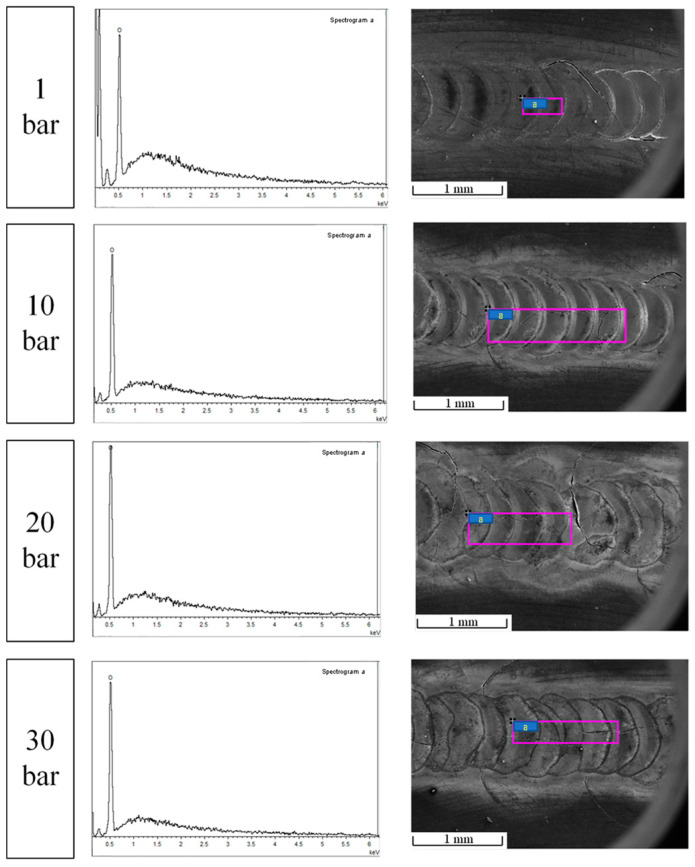
Surface energy spectrum results of beryllium laser melting (“a” represents the corresponding analusis area).

The test results of the surface energy spectrum of the beryllium laser melting channel under different pressures are shown in Figure 5.

Because of the strong oxidation property of beryllium, an oxide-protective film can be formed on the surface of beryllium in air, which is stable at room and high temperatures. Because the X-ray signal generated by beryllium is excessively weak, it is easily covered by the back and bottom peaks, meaning that beryllium has a high X-ray penetration rate (almost transparent) [5]; thus, it is difficult to analyze on the energy spectrum and cannot be identified using EDS. Therefore, the O element in the energy spectrum accounts for 100%. As shown in the figure, when the ambient pressure was 1 bar, there was an unrecognized peak on the left side of the O element.

### 4.3. Cross-Section Shape and Microstructure of Solidification Pools

**Figure 6:**
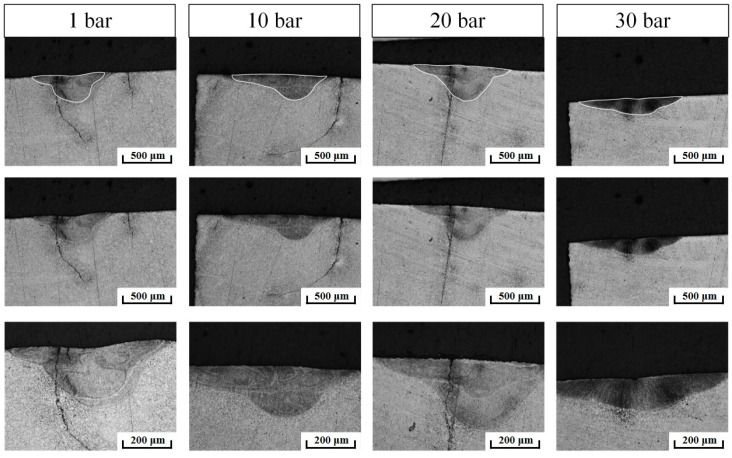
Cross-section shape of laser melting channel of beryllium under different ambient pressures.

The metallographic structure of the laser melting channel section of beryllium under different pressure environments is shown in Figure 6. The “small hole” trend appeared at low pressures, and it abruptly weakened at 30 bar. When the ambient pressure reached 30 bar, the bath width increased to its widest, but the depth did not increase. For the lasered direct-deposition process, a wide and shallow molten pool is more conducive to the stability of the deposition process; therefore, an appropriate positive-pressure environment can be a positive factor.

## 5. Discussion

### Evolution of the Laser Melting Process

For pulsed laser processing, the highest temperature of the molten pool is higher than the boiling point of the material, resulting in the generation of metal vapor. When the metal vapor continues to absorb a large amount of energy and ionize, plasma is produced. The mixture of metal vapor and ionized plasma is called a “plasma plume” [25].

Beryllium will form a passivation oxide layer on the surface at room temperature, which has a certain protective effect, where the oxidation corrosion is weak. When the temperature rises to about 500 °C, the oxidation of beryllium will intensify, resulting in loose particles and holes. During laser processing at 1 bar (normal pressure), the temperature of the molten pool exceeds 1000 °C, and the metal vapor erupts violently [26], which makes the oxidation corrosion very serious. As shown in Figure 3, under normal pressure, the beryllium matrix is basically not observed in the solidified molten pool. Therefore, in an atmospherically inert gas atmosphere, the beryllium surface becomes oxidized, and the oxidation is severe. This oxidation primarily includes oxidation steam and direct oxidation near the molten pool.

Because a high-pressure environment limits the diffusion of metal vapor [27,28], when the ejection and expansion of a metal vapor are limited under high pressure, the likelihood of the occurrence of the reaction between metallic particles and residual oxygen in the pressure vessel is also limited. Additionally, the formation of oxides is also affected by the oxygen concentration. The residual amount of oxygen remains unchanged, the pressure increases, and the more argon is charged, the more serious the dilution of oxygen, which leads to a further minimization of the oxidation reaction. Consequently, the solidified molten pool processed at high pressure appears shiny as it is not covered by oxide particles.

Therefore, the formation of oxides is related to both the environmental pressure and laser energy density. When the environmental pressure is high, and the energy density is low, the oxidation phenomenon can be inhibited, meaning that favorable conditions for oxidation are provided. As shown in Figure 3, when the ambient pressure reached 10 bar or above, the surface oxidation was basically eliminated.

Because of the violent eruption of metal vapor at low pressure, the surface of the molten pool fluctuates before solidification, which produces ripples on the surface of the molten pool, creating a shape of the molten pool approximate to a regular circle. As environmental pressure increases, the ejected metal vapor is limited to a small area close to the upper surface of the molten pool and cannot disperse, forming a local violent flow of air on the upper surface of the molten pool [27]. When the air flows, it disturbs the original shape of the molten pool and changes the original uniform flow and heat transfer of the surface metal liquid; thus, the contour of the molten pool becomes gradually irregular.

Some researchers have indicated that to overcome the generation of cracks in the laser welding of beryllium, the melting of beryllium must be reduced. This is an effective method of reducing crack sensitivity [29]. This is because the pressure affects the melting point of the object in which the crack can be eliminated by increasing the ambient pressure. We often state the melting point at atmospheric pressure. The melting point of a substance is usually referred to as the situation at an atmospheric pressure. If the pressure changes, the corresponding melting point also changes. Generally, the melting point increases with an increase in pressure. In other words, the boiling point of the molten pool increases under high pressure, resulting in less base metal melting under the same laser energy, thus decreasing the generation rate of cracks. Additionally, the plasma plume in a high-pressure environment also causes scattering, reflection, and other effects on the laser energy input. The results indicated that the surface of the molten pool was heated evenly, the shape of the molten pool was wide and shallow, the deposition process was stable, and the cracking was restrained.

The main impurity phase of beryllium is BeO, which is a hexagonal wurtzite crystal categorized as an ionic compound crystal that is difficult to deform, and its occurrence is equivalent to a crack or hole defect [30]. The increase in environmental pressure can effectively eliminate the oxidation of beryllium in laser processing, significantly reduce the content of BeO in the sample, and reduce the location of stress concentration (defects occur), thus decreasing the trend of cracks.

Under high pressure, plasma and metal vapor are difficult to disperse and converge on the upper surface of the molten pool, which prevents the formation of “small holes” in the molten pool. Additionally, the plasma plume gathered on the upper surface of the molten pool can provide radiation heat there while absorbing laser energy, which increases the absorption of the laser energy on the surface of the molten pool, thus increasing the radial heat of the molten pool [27] and increasing the width of the molten pool.

## 6. Conclusions

In this study, the influence of ambient pressure on the single-pulse laser processing of beryllium was explored by building an inert gas pressure atmosphere-assisted laser processing device. The wavelength of a Nd:YAG laser as the incident laser was 1.06 μm. The experiment was performed in a pressure vessel with argon at pressures ranging from 1 to 30 bar. The results are outlined as follows:

1. Although the laser absorptivity of pure beryllium is relatively low, it can absorb the laser and form a molten pool. The contour area of the upper surface of the molten pool is approximately 80% of that of 304 stainless steel under the same energy input;
2. Beryllium is highly reactive. Under vacuum or low pressure, owing to the violent eruption of metal vapor, beryllium reacts with the residual oxygen in the environment to form oxides and falls on the surface and around the molten pool. When the ambient pressure is higher than 10 bar, oxidation is eliminated. A high-pressure environment and low energy density can eliminate oxidation;
3. Beryllium cracks easily during laser processing. As pressure increases, the surface cracking of beryllium changes from “divergent” to “shrinkage”, which demonstrates that the high-pressure environment has the tendency to eliminate the cracks in the process of beryllium laser processing;
4. The ambient pressure affects the laser-processing melting of beryllium. It is easy to form a greater penetration at low pressure, exhibiting the trend of “small holes”. When the pressure increases, the formation of “small holes” is inhibited. When the ambient pressure reaches 30 bar, the generation of “small holes” is completely suppressed, and the width of the molten pool increases to the widest. For the direct deposition process, a wide and shallow molten pool is more conducive to the stability of the deposition process. Therefore, a suitable barotropic environment can have a positive effect.

## Figures and Tables

**Table 1:** Laser parameters and motion table parameters in pulsed laser wire-feeding deposition.

Parameters	Single-Pulse Energy	Current of Laser Power Supply	Pulse Width	Frequency	Scanning Speed	Defocusing Amount
Value	55 J	240 A	4 ms	3 Hz	25 mm/min	−5 mm

**Table 2:** Schematic of the detection methods used in different detection positions.

Position in the Molten Pool	Illustrations	Observation Methods
Surface	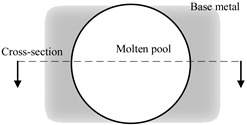	LSCM SEM/EDS
Cross-section	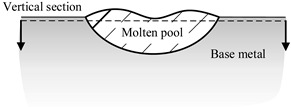	LSCM

## Data Availability

Not applicable.
